# Hippocampal morphology and cognitive functions in community-dwelling older people: the Lothian Birth Cohort 1936

**DOI:** 10.1016/j.neurobiolaging.2016.12.012

**Published:** 2017-04

**Authors:** Maria del Carmen Valdés Hernández, Simon R. Cox, Jaeil Kim, Natalie A. Royle, Susana Muñoz Maniega, Alan J. Gow, Devasuda Anblagan, Mark E. Bastin, Jinah Park, John M. Starr, Joanna M. Wardlaw, Ian J. Deary

**Affiliations:** a Centre for Cognitive Ageing and Cognitive Epidemiology, University of Edinburgh, Edinburgh, UK; b Department of Neuroimaging Sciences, Centre for Clinical Brain Sciences, University of Edinburgh, Edinburgh, UK; c Scottish Imaging Network, a Platform for Scientific Excellence (SINAPSE) Collaboration, Edinburgh, UK; d Department of Psychology, University of Edinburgh, Edinburgh, UK; e School of Computing, Korea Advanced Institute of Science and Technology (KAIST), Daejeon, South Korea; f Department of Psychology, Heriot-Watt University, Edinburgh, UK

**Keywords:** Hippocampus, Memory, Aging, Morphology, Mesh models, Intelligence

## Abstract

Structural measures of the hippocampus have been linked to a variety of memory processes and also to broader cognitive abilities. Gross volumetry has been widely used, yet the hippocampus has a complex formation, comprising distinct subfields which may be differentially sensitive to the deleterious effects of age, and to different aspects of cognitive performance. However, a comprehensive analysis of multidomain cognitive associations with hippocampal deformations among a large group of cognitively normal older adults is currently lacking. In 654 participants of the Lothian Birth Cohort 1936 (mean age = 72.5, SD = 0.71 years), we examined associations between the morphology of the hippocampus and a variety of memory tests (spatial span, letter-number sequencing, verbal recall, and digit backwards), as well as broader cognitive domains (latent measures of speed, fluid intelligence, and memory). Following correction for age, sex, and vascular risk factors, analysis of memory subtests revealed that only right hippocampal associations in relation to spatial memory survived type 1 error correction in subiculum and in CA1 at the head (*β* = 0.201, *p* = 5.843 × 10^−4^, outward), and in the ventral tail section of CA1 (*β* = −0.272, *p* = 1.347 × 10^−5^, inward). With respect to latent measures of cognitive domains, only deformations associated with processing speed survived type 1 error correction in bilateral subiculum (*β*_*absolute*_ ≤ 0.247, *p* < 1.369 × 10^−4^, outward), bilaterally in the ventral tail section of CA1 (*β*_*absolute*_ ≤ 0.242, *p* < 3.451 × 10^−6^, inward), and a cluster at the left anterior-to-dorsal region of the head (*β* = 0.199, *p* = 5.220 × 10^−6^, outward). Overall, our results indicate that a complex pattern of both inward and outward hippocampal deformations are associated with better processing speed and spatial memory in older age, suggesting that complex shape-based hippocampal analyses may provide valuable information beyond gross volumetry.

## 1 Introduction

The role of the hippocampus in cognitive processes, particularly in a variety of memory functions (including verbal encoding and retrieval, spatial navigation, and working and short-term memory), is well studied (Jonides et al., 2008). Via its dense connections with other important cerebral loci, its processes also support cognitive abilities more generally (Rubin et al., 2014). Evidence that hippocampal volume is related to memory performance is most prevalent among populations which show age-related or pathological hippocampal atrophy (van Petten, 2004). Lower hippocampal volumes among patients with Alzheimer's disease (AD) mild cognitive impairment (MCI), and depression are associated with poorer verbal and non-verbal/spatial memory scores (Bonner-Jackson et al., 2015, Grundman et al., 2003, Hickie et al., 2005, Peruzza Marchiani et al., 2008; see van Petten, 2004 for a review pre-2004). Similarly, among nonpathological samples of older adults, differences in hippocampal volume are related to poorer memory performance, mainly quantified using verbal recall tasks (Aribisala et al., 2014, den Heijer et al., 2012, Raz and Rodrigue, 2006, Ystad et al., 2009). Reduction in hippocampal volume has also been linked with poorer cognitive performance in a variety of cognitive domains in addition to memory, such as fluid intelligence (Reuben et al., 2011) and processing speed (Papp et al., 2014). However, a study in a group of 518 older adults (den Heijer et al., 2010) from a population-based cohort reported that the rate of decline in hippocampal volume over 10 years was related specifically to verbal memory but not to general indicators of cognitive performance (e.g., mini-mental state examination score) or measures of executive function.

Aside from potential confounders of sample size, age, gender, and vascular risk factors (Bender et al., 2013, Cahill, 2006, Shing et al., 2011, Ystad et al., 2009), other possible reasons for the somewhat inconsistent evidence of the association between total hippocampal volume and cognitive performance might be that different hippocampal regions are differently sensitive to age, and/or to different cognitive tests (Hackert et al., 2002) and exhibit distinct shrinkage/enlargement effects that may compensate overall volumetric variations in this structure. One approach to test this theory has been to measure the volumes of specific hippocampal subfields, but there is no consensus on a single segmentation protocol (Adler et al., 2014, Shing et al., 2011). In addition, 1.0-mm isotropic voxels obtained at 1.5 T, commonly used by many MR protocols, produce images too coarse to reliably delineate hippocampal subfields. Acquisition protocols at higher magnetic fields of ∼0.4 × 0.4 mm or less in-plane resolution of the hippocampal region have been used by studies specifically aiming at the study of this structure (Adler et al., 2014). But even with optimal acquisition methods, anatomical delineation of hippocampal subfields is challenging. As subfield morphology is subject to individual differences, using atlas-bases measures for identification of fine-grained details is inconsistent with routine clinical image acquisition protocols. An alternative method has been to examine hippocampal shape morphology—which does not consider subfield boundaries established a priori. Analyses assessing the hippocampus in this way have reported age-related inward deformations in the hippocampal head and subiculum, regardless of age-related hippocampal volume reduction (Yang et al., 2013), and sub-regional associations with other cognitive domains, in addition to memory, across the whole lifespan.

A consistent finding from across studies that relate hippocampal morphology with cognitive measures is the association of cognitive performance with deformations in the cornu ammonis (CA1) at the hippocampal head. For example, on 383 data sets extracted from the Alzheimer's Disease Neuroimaging Initiative database (http://adni.loni.usc.edu/), the anterior hippocampus and the basolateral segment of the amygdala showed a deformation inward in AD and MCI patients with respect to cognitively normal individuals, consistent with associated memory deficits on this population (Qiu et al., 2009). In 137 individuals of 18–86 years of age, a lengthening of the antero-posterior axis left hippocampus was prominently associated with working memory performance across the adult lifespan (Voineskos et al., 2015). A study on 103 MCI subjects (Costafreda et al., 2011) revealed an atrophy pattern associated with rapid cognitive deterioration in mini-mental state examination scores and verbal memory that showed initial degeneration in the anterior part of CA1. Another study also showed a significant decrease in the volumes of CA1 and subiculum subfields in AD compared with cognitively normal individuals (Perrotin et al., 2015). Yet, in spite of the importance of the hippocampus in healthy and pathological aging, a comprehensive analysis of multidomain cognitive associations with hippocampal deformations among a large group of cognitively normal older adults is currently lacking.

Here, we extend our previous pilot analysis conducted on a small subsample (n = 51) of an age-homogeneous (73 years) cohort of cognitively normal older individuals (Kim et al., 2015) to examine associations between hippocampal morphology and a wider range of cognitive functions, both at the level of cognitive domains and with respect to individual subtests, on a sample that is 13 times larger. While examining the possibility of added value in using hippocampal shape analysis in conjunction with volumetry, the aim of the study is to explore hippocampal shape associations between a wide range of cognitive functions. Such associations may indicate loci particularly sensitive to the cognitive functions we evaluate and may also be coincident with loci reported in other studies to be vulnerable to the neuropathologies of aging (Tang et al., 2016). By exploring these associations on a larger sample, we aim to answer the following questions: (1) is the inward deformation on the hippocampal head reported by other studies (Perrotin et al., 2015, Qiu et al., 2009, Ta et al., 2012) associated with reduced general cognitive functioning on a cognitively normal aging population and/or related to their childhood intelligence? (2) In nondemented older individuals, is regional hippocampal morphology associated with other cognitive functions or only with memory as reported elsewhere (Hackert et al., 2002)? In line with the studies referenced above, we hypothesize that in this cohort of septuagenarian individuals' hippocampal morphology, and specifically lateral deformations on the surface of the hippocampal head, will be associated with specific memory ability and also with broader cognitive domains. Given prior evidence in the hippocampus (Tang et al., 2016) and associations between earlier life intelligence and other MRI phenotypes in this cohort (e.g., Cox et al., 2016, Field et al., 2016), we further hypothesize that precursors of these deformations could be found at childhood.

## 2 Materials and methods

### 2.1 Participants

The Lothian Birth Cohort 1936 (LBC1936; Deary et al., 2007) provided the sample for the present analysis. The LBC1936 is a large study of older community-dwelling adults, mostly living in the Edinburgh and Lothians area of Scotland, all of whom were born in 1936 and most of whom participated in the Scotland Mental Survey of 1947 at age 11 years. At ∼70 years, study participants (N = 1091) underwent an initial wave of cognitive and physical testing, from 2004–2007. Approximately 3 years later, 866 underwent a second wave of cognitive tests at mean age 72.8 years (SD = 0.7; Deary et al., 2012) which also involved an optional brain MRI scan. All data in the current study are taken from this second wave. The brain scan was undertaken by 700 subjects, yielding 681 participants with useable MRI data. Of these, 654 participants (345 women and 309 men) who also had complete cognitive data, were the subject of the present analysis. The Multi-Centre Research Ethics Committee for Scotland (MREC/01/0/56), Scotland A Research Ethics Committee (07/MRE00/58) and Lothian Research Ethics Committee (LREC/2003/2/29) approved the use of the human subjects in this study; all participants provided written informed consent and these have been kept on file.

### 2.2 Cognitive abilities

Participants who attended the second wave of the LBC1936 study also underwent a number of cognitive tests. These included 6 subtests from the Wechsler Adult Intelligence Scale (WAIS-III^UK^; Wechsler, 1998a): symbol search, digit symbol, matrix reasoning, letter-number sequencing, digit span backward, and block design, alongside 6 subtests from the Wechsler Memory Scale III^UK^ (WMS III^UK^; Wechsler, 1998b): Logical memory immediate and delayed recall, spatial span forward and backward, and verbal paired associates (1st and 2nd recalls). They also provided measures of simple and 4-choice reaction time and inspection time (Deary et al., 2007). These were used to examine associations with the hippocampus for both memory subtests, and for cognitive domains (see Section 2.7). Cognitive ability at age 11 was assessed using the Moray House Test IQ score from the Scottish Mental Survey of 1947, which is considered a good measure of general intelligence (Deary et al., 2007, Scottish Council for Research in Education, 1949).

### 2.3 Vascular risk factors

Continuous measures of body mass index, average systolic and diastolic blood pressure, and glycosylated hemoglobin, were obtained (as per Deary et al., 2007, Deary et al., 2012). Also, at wave 2, participants provided information on vascular and health factors during a medical interview. They were asked whether they had received a diagnosis of hypertension, high cholesterol, or diabetes, about their history of cardiovascular disease, previous strokes, and their smoking status (current, ex- or never). Presence of each self-reported factor was coded as 1 (0 denoted absent) except smoking status (2, 1, 0). An aggregate score of contemporaneous vascular risk was derived from these factors and the presence (1)/absence (0) of old infarcts identified on the MRI scan (as per Valdés Hernández et al., 2013).

### 2.4 MRI acquisition

MRI scans were acquired using a GE Signa Horizon 1.5-T HDxt clinical scanner (General Electric, Milwaukee, WI, USA) operating in research mode using a self-shielding gradient set with maximum gradient of 33 mT/m and an 8-channel phased-array head coil. The imaging protocol is fully described elsewhere (Wardlaw et al., 2011). For this particular study, we used data obtained from processing coronal T1-weighted volume scans acquired with a 3D inversion recovery prepared fast gradient echo sequence (TR/TE/TI = 9.7/3.984/500 ms, flip angle α = 8°, bandwidth 15.63 kHz, voxel size 1 × 1 × 1.3 mm^3^, and field of view in the acquisition plane 256 × 256 mm^2^).

### 2.5 MRI analysis: structural segmentations

Hippocampal shape models were generated from binary masks obtained semiautomatically from the T1-weighted volumes. First approximations of left and right hippocampal segmentations were obtained from an automated pipeline that uses tools from the FMRIB Software Library version 4.1 (Oxford, UK; http://www.fmrib.ox.ac.uk/fsl/), and an age-relevant template (Farrell et al., 2009), followed by visual inspection and manual correction when required using Analyze 10.0 software (Mayo Clinic, Rochester, MN, USA; www.analyzedirect.com), and saved as binary masks as per previous publications (Aribisala et al., 2014, Wardlaw et al., 2011). Semi-automated measurements of intracranial volume (ICV; contents within the inner skull table including brain tissue, cerebrospinal fluid, veins, and dura; Valdes Hernandez et al., 2012) were used for normalization.

### 2.6 MRI analysis: shape model analysis

The hippocampal shape modeling and analysis of the local deformations are done in 4 steps: (1) construction of the sample-relevant deformable template model (DTM) of the target structure (e.g., left and right hippocampi); (2) template deformation and construction of the individualized shape models; (3) surfaces' alignment; and (4) computation of the local deformations. Full explanation can be found at http://cgv.kaist.ac.kr/brain/, and the toolbox that implements each step can be accessed from http://www.nitrc.org/projects/dtmframework/. In principle, hippocampal binary masks were input to a non-rigid shape modeling framework (i.e., DTM framework) that uses a progressive model deformation technique built-up on a Laplacian surface representation of multi-level neighborhood and flexible weighting scheme (Kim et al., 2015). Briefly, the surface of a 3D model that encodes the generic shape characteristics of all hippocampi from the sample as a triangular mesh is non-rigidly deformed in a large-to-small scale to allow recovery of the individual shape characteristics, while minimizing the distortion of the general model's point distribution. This surface deformation is achieved through an iterative process that, at each iteration, decreases a rigidity weight α and the level of neighborhood in a step-wise way together with the magnitude of the displacement of each vertex. At early iterations, the generic 3D model deforms more largely to reproduce the large shape features of the hippocampus by propagating the external force, guiding each vertex of the general model to the closest image boundary, across the surface. In the iteration process, when the general model is not deformed anymore by the balance between the external and internal forces, the rigidity and the level of neighborhood are gradually diminished so that the model deforms at smaller regions to reproduce local shape details. To preserve the surface quality and diminish the effect that rough boundaries and noise in the binary masks could pose to the shape analyses, a rotation and scale-invariant transformation that constrains the vertex transformations only to rotation, isotropic scale, and translation is applied afterward. This helps regularizing the individual vertex transformations to those of the neighboring vertices using them as reference.

The sample's right and left hippocampal DTM are constructed by applying marching cubes, mesh smoothing and mesh resampling methods to hippocampal “atlases” obtained from averaging the coregistered binary masks from all participants' hippocampi (Kim et al., 2015). Our left and right hippocampal DTM are triangular meshes of 4002 vertices each. The quality of the modeling process (steps 1 and 2, explained above) was evaluated using 3 metrics: (1) the volumetric similarity index (i.e., dice coefficient; Lee, 1945, Zhou et al., 2004) calculated as the sum of true positives and negatives divided by the sum of true and false positives and negatives; (2) the mean; and (3) maximum distances between the points of the individualized surface (i.e., mesh) models and the corresponding boundaries of the binary masks. The first metric is calculated after converting the individualized surface models to binary images as the sum of the true positives and negatives divided by the sum of true and false positives and negatives. True positives are the voxels of this ‘mesh-to-binary’ converted image that are coincident with those of the binary mask used as input in the modeling. In turn, true negatives are those which were not part of either of the binary images. The third metric is known in the technical literature as fiducial localization error (Fitzpatrick et al., 1998). When these metrics suggested that the precision of the modeling method was more than half the voxel size, the modeling process was re-run with different values of the rigidity parameter, number of iterations, neighborhood rings, and offsets until a good fit was achieved.

After the 4002-vertex surface mesh model was fit to each hippocampal binary mask, all meshes were coregistered and scaled using the individuals' ICV, and an average mesh (i.e., a sample-specific “template”) was generated (step 3). This “template” mesh was then aligned back to each individual mesh (i.e., one-by-one transformations to “native” space) to calculate the deformation of each point (i.e., mesh vertex) from each hippocampus with respect to the correspondent point in the sample-specific “template”. This last step (step 4) generated 2 text files (1 for each hippocampus) with the values of the deformation vectors for each point of each data set.

### 2.7 Statistical analysis

Cognitive test scores were examined both at the subtest and domain level. For the subtests, we examined spatial memory (sum of spatial span forward and backward), verbal memory (first unrotated component of a PCA from immediate and delayed parts of both logical memory and verbal paired associates; loadings all >0.83, accounting or 71.8% of the variance), and scores on digit span backward, and letter-number sequencing. At the domain level, we used PCA to create 3 latent variables representing the cognitive domains of memory (g-memory), information processing speed (g-speed), and the hierarchically superordinate domain of general fluid intelligence (*g*). This data reduction approach is common for deriving a latent, underlying construct which is free from item-level measurement error and test-specific variance (e.g., Penke et al., 2012). The cognitive tests, loadings, and proportion of variances explained by the first unrotated component in each domain are shown in Supplementary Table 1. Further details on the cognitive tests are reported in 2 open-access protocol papers (Deary et al., 2007, Deary et al., 2012).

Associations between cognitive variables and hippocampal morphology were evaluated with multiple regression using MATLAB R2015a (http://uk.mathworks.com). Initially, we explored how much cognitive function in older age can be explained by local deformations. This model used the deformation vector at each point of the hippocampal triangular meshes as the predictor (i.e., independent variable) and each cognitive subtest variable as the response (i.e., dependent variable). We then investigated these associations at the level of the cognitive domains *g*, g-memory, and g-speed. Next, we explored how much local hippocampal surface deformations in older age depended on childhood intelligence (i.e., age 11 IQ) and used the latter as predictor.

Age in days at the time of the scanning, gender, and vascular risk score were used as covariates in all models. We also ran supplementary analyses for hippocampal volume (for comparative purposes with morphological results). We calculated correlations between hippocampal volume (raw and corrected for brain size), cognitive and vascular risk variables (Pernet et al., 2013), and linear regressions using the same age, gender, and vascular risk measures as for the morphological analysis.

Given the well-known vascular substrate of neurodegeneration and cognitive impairment (Jellinger, 2013), and the links between vascular risk factors and cognitive decline (Duron and Hanon, 2008), we explored whether vascular risk factors were directly associated with local hippocampal shape deformations, and if there were any mediating effects in the associations between hippocampal deformations and cognitive function (if present). The beta coefficients and *p*-values for each of the 4002 points were mapped on the reference (i.e., “template”) surface to display the deformation patterns in relation to each cognitive variable. Standardized *β*s are reported throughout, and *p*-values were corrected for multiple comparisons using false discovery rate (FDR; Benjamini and Hochberg, 1995) as recommended by Glickman et al. (2014). Finally, we ran a sensitivity analysis to account for the presence of participants who may be exhibiting pathological aging. Though all participants were free from dementia diagnosis at initial recruitment (∼3 years before MRI), we identified those who had either reported a dementia diagnosis or had a mini-mental state examination (MMSE; Folstein et al., 1975) score <24 at either wave 2 or wave 3 of the study. A dichotomous covariate reflecting whether either criterion was fulfilled (n = 22) was included in sensitivity models (which also included age, gender, and vascular risk), and the loci and magnitudes of associations between cognitive scores and hippocampal morphology were compared with previous model outputs.

## 3 Results

Characteristics of study participants are shown in Table 1. Participants' mean total hippocampal volume was 6429.10 (SD = 867.29) mm^3^, and associations between hippocampal volumes and study variables are shown in Table S2. Participants attending MRI did not significantly differ from those who only attended cognitive testing across any memory subtests or at the level of any cognitive domains (*t* ≤ 1.534, *p* ≥ 0.127).

### 3.1 Quality of the hippocampal shape modeling process

**Fig. 1:**
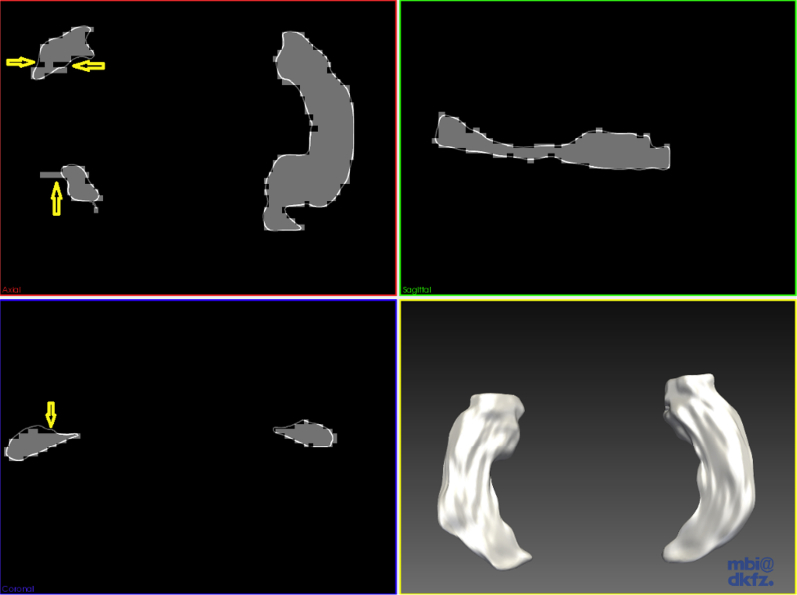
Shape model of left and right hippocampi of a data set where the binary mask has few voxels missing/out of the contour of the shape model (arrowed). The fiducial localization error (not shown) was 8.07 mm and the mean distance between the surface mesh model (represented in white) and the binary mask (gray) was 0.51 mm. The axial (top left), sagittal (top right), and coronal (bottom left) views were selected to show the fitness of the mesh model to the binary masks of left and right hippocampi, the representativeness of the hippocampal shape details by the model, and the compensation of voxelization effects (Image generated with MITK v2013.06.0 http://www.mitk.org/).

Median Dice coefficient values (i.e., spatial volumetric similarity index) were 0.96 (IQR 0.027) for both (i.e., left and right) hippocampi. Median hippocampal surface-binary mask mean differences were 0.22 mm (IQR 0.21) for left hippocampi and 0.29 mm (IQR 0.38) for the right, indicating that the surface models accurately reproduced the hippocampal shape details. The median fiducial localization error for the left hippocampus was 4.20 mm (IQR 7.22), and for the right hippocampus it was slightly higher 6.91 mm (IQR 7.01). Further investigation revealed that the latter, which measures the maximum distance between the surface model and the binary mask, was high due to rough boundaries on the binary masks arising from voxelization (Fig. 1) and the presence of small T1-weighted hypointense cavities (Viksne et al., 2015). Although their nature is unknown, these cavities are normal features of aging: some of them may represent a diffuse vascular process with adverse local effects and/or proxies for larger volumes of infarcts or mild or severe diffuse damage.

### 3.2 Associations between measures of memory subtests and hippocampal morphology

**Fig. 2:**
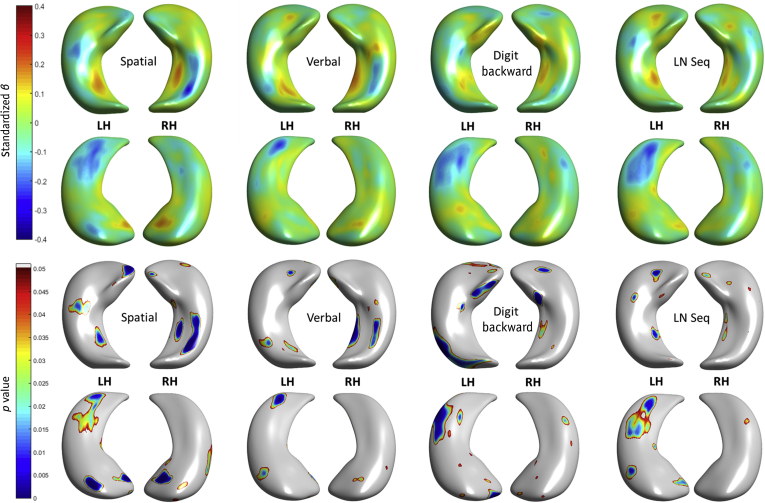
Regional associations between hippocampal shape deformations and specific memory tests, correcting for age, sex, and vascular risk. Coefficient estimates *β*s (top 2 rows: superior and inferior views) and *p*-values (bottom 2 rows also superior and inferior views) are shown. Abbreviations: LH, left hippocampi; LN Seq, letter-number sequencing; RH, right hippocampi.

**Fig. 3:**
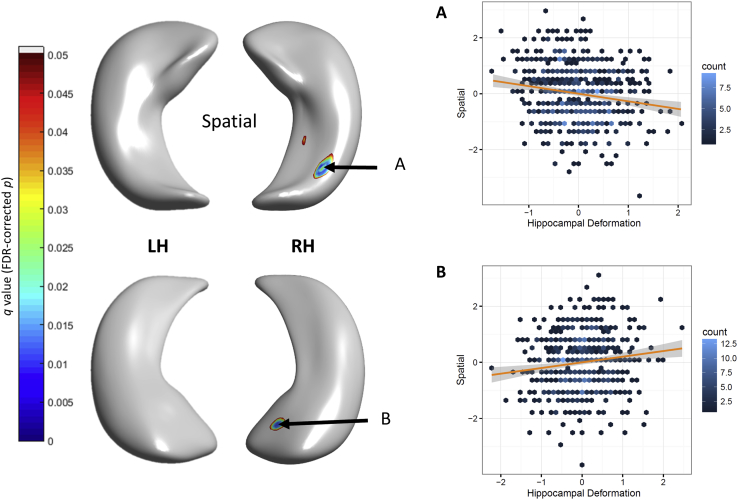
FDR-corrected significant associations (with 95% CIs) between hippocampal deformation and spatial span performance. Test statistics are shown for cluster centers. (A): ventral tail section of CA1, standardized *β* = −0.272, *p* = 1.347 × 10^−5^ (inward); (B): subiculum and CA1 at the head, standardized *β* = 0.201, *p* = 5.843 × 10^−4^ (outward). Abbreviations: CA1, cornu ammonis; CI, confidence interval; FDR, false discovery rate.

Regional differences in hippocampal morphology with respect to measures of specific memory subtests are shown in Fig. 2 (coefficient estimates *β* and uncorrected significance). The standard errors of all cognitive models are shown in Supplementary Fig. 1. At uncorrected significance levels (*p* < 0.05), better performance across 4 measures (verbal, spatial, letter-number sequencing, and digit span backward) was associated with both inward and outward hippocampal deformations with respect to the template (i.e., representing the mean hippocampal shape of the sample). Outward deformations at the bilateral right medio-ventral tail and bilateral inward deformations at the dorsal tail were consistently associated with superior performance across tests, though with differing magnitudes. Only right hippocampal associations involving extreme deformation patterns in relation to spatial span performance survived FDR correction (Fig. 3); this was in subiculum and CA1 at the head (*β* = 0.201, *p* = 5.843 × 10^−4^, outward), and in the ventral tail section of CA1 (*β* = −0.272, *p* = 1.347 × 10^−5^, inward).

### 3.3 Associations between general cognitive measures and hippocampal morphology

**Fig. 4:**
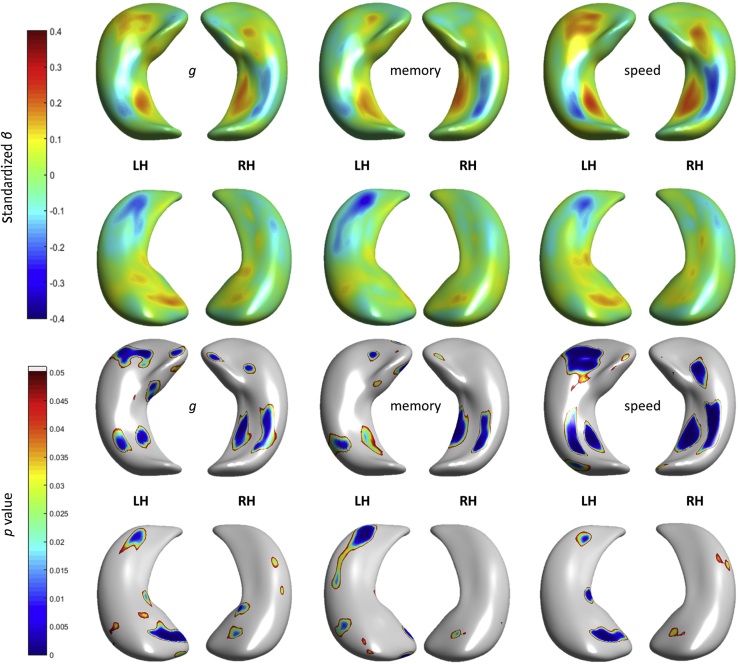
Regional associations (top row) between hippocampal shape deformations and general cognitive measures, correcting for age, sex, and vascular risk. Coefficient estimates *β*s (top 2 rows showing superior and inferior views) and *p*-values (bottom 2 rows also showing superior and inferior views) are shown. Abbreviations: LH, left hippocampi; RH, right hippocampi.

**Fig. 5:**
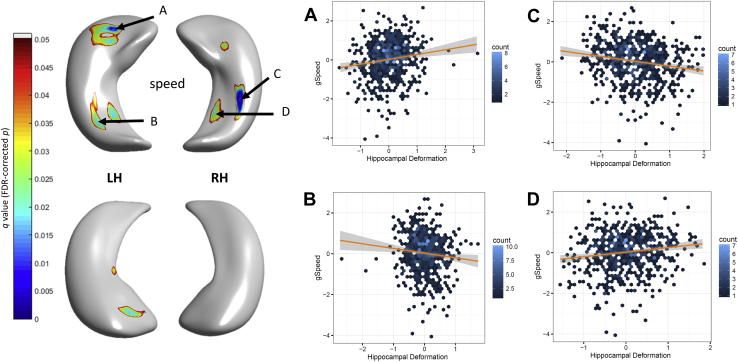
FDR-corrected significant associations (with 95% CIs) between hippocampal deformation and processing speed performance. Test statistics using standardized coefficient estimates are shown for cluster centers A: *β* = 0.248, *p* = 1.370 × 10^−4^; B: *β* = −0.231, *p* = 0.0061; C: *β* = −0.242, *p* = 3.451 × 10^−6^; D: *β* = 0.227, *p* = 2.073 × 10^−4^.

Regional differences in hippocampal morphology with respect to general cognitive factors are shown in Fig. 4 (coefficient estimates *β* and uncorrected *p* values). Memory domain scores broadly replicated the inward and outward deformation patterns with respect to the mean surface of the sample across memory subtests, outlined above. Bilateral deformations on CA1 at the hippocampal head and dorsal tail, at the junction between hippocampal head and tail and subiculum were associated with processing speed. A modest and nonsignificant association with general cognitive abilities (g) was observed at the dorsal head of left hippocampus (CA1). After applying FDR correction, only associations involving regions with extreme deformation patterns associated with processing speed survived (Fig. 5): in subiculum (*β* = 0.247, *p* = 1.369 × 10^−4^, outward), in the ventral tail section of CA1 (*β* = −0.230, *p* = 0.0061, inward), at the anterior-to-dorsal region of the head (*β* = 0.199, *p* = 5.220 × 10^−6^, outward) for left hippocampus; and in subiculum (*β* = 0.227, *p* = 2.073 × 10^−4^, outward) and in the ventral tail section of CA1 (*β* = −0.242, *p* = 3.451 × 10^−6^, inward) for right hippocampus.

### 3.4 Associations between hippocampal morphology in older age and childhood intelligence

**Fig. 6:**
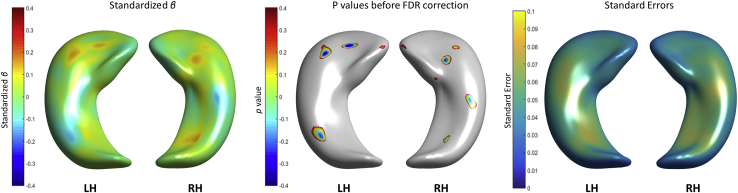
Associations between hippocampal shape deformations and age 11 IQ (left), significance (*p*-values) before correcting for multiple comparisons (middle) and standard errors (right). Abbreviations: LH, left hippocampi; RH, right hippocampi.

Childhood intelligence, represented by age 11 IQ, did not predict hippocampal shape deformations in older age. Fig. 6 shows that the model fitted the data (very small standard error values, i.e., <0.1), but no associations survived FDR correction.

### 3.5 Associations between hippocampal morphology at old age and vascular risk factors

Body mass index and self-reported vascular risk factors (hypertension, hypercholesterolemia, diabetes, history of cardiovascular disease, previous strokes, and smoking status all summed on a total score) exhibited nominal uncorrected associations with inward deformations at the lateral head of each hippocampi (Supplementary Fig. 2). However, these associations did not survive FDR correction. Therefore, there was no basis from which to conduct formal mediation analyses to inquire whether vascular risk factors mediated any associations between hippocampal shape and cognitive functions. Of note, an additional evaluation of the associations between cognitive variables and hippocampal morphology excluding the vascular risk factor score as a covariate did not show difference in the graphic representation of the results presented above.

### 3.6 Sensitivity analysis

Accounting for dementia diagnosis among participants did not significantly alter the loci or magnitude of the reported effects. For example, maximal cluster peaks for speed in the left hippocampus changed from *β* = −0.231 to −0.218 and 0.248 to 0.251, and in the right hippocampus from *β* = −0.242 to −0.234 and 0.227 to 0.221. For spatial, from *β* = 0.201 to 0.189, and −0.272 to −0.247. All values still remained significant following FDR correction.

### 3.7 Supplementary volumetric analysis

Supplementary analyses for hippocampal volume are shown in Table S2 (bivariate correlations) and Table S3 (multivariate regressions). When modeled with cognitive tests covarying for age, sex, and vascular measures, raw volumes (predominantly on the left side), were associated with verbal memory (*β* = 0.115, *p* = 0.006), digit backward (*β* = 0.120, *p* = 0.004), and letter-number sequencing (*β* = 0.108, *p* = 0.010). Total hippocampal volume was also significantly associated with the cognitive domains *g* (*β* = 0.126, *p* = 0.002) and memory (*β* = 0.137, *p* = 0.002). However, while these results survived FDR correction for multiple comparisons, adjusting the hippocampal volumes for brain size attenuated all associations to nonsignificance.

## 4 Discussion

Here, we report that associations between hippocampal characteristics and cognitive abilities show hippocampal-wide volumetric effects alongside complex and regionally specific morphological deformations. We found associations between regional shape deformations in the right hippocampus and spatial memory, and between processing speed and a more distributed set of bilateral regions. Notably, these 2 cognitive measures (spatial memory and processing speed) did not show any associations with hippocampal volume, indicating that volumetric and morphological analyses provide complimentary information on a brain formation which is intimately involved in multiple cognitive functions. In particular, our results highlight the importance of the CA1 subfield in cognitive performance among this group of healthy older adults, in agreement with other studies (Costafreda et al., 2011, Voineskos et al., 2015). While a previous study on a group of 104 healthy young adults reported a complex pattern of inward and outward hippocampal deformations with respect to the mean hippocampal shape of the sample being associated with measures of spatial intelligence and spatial memory but not with processing speed (Colom et al., 2013), our contrasting findings in this (much older) cohort may be due to the increased proportion of shape variance due to differential age effects, which may subsequently account for more variance in cognitive performance. Processing speed is well known to be highly sensitive to aging (Raz and Rodrigue, 2006), but current research indicates a central role for white matter in processing speed in older age (Penke et al., 2012). Nevertheless, hippocampal volume has been reported to contribute uniquely to processing speed beyond white matter hyperintensities (Papp et al., 2014), suggesting that hippocampal deformations may provide unique information about cognitive variability in older populations.

The main pyramidal layers of the hippocampus are found predominantly in CA1, along with CA3 and the subiculum. Given that these layers receive axonal projections from the perforant path (a major hippocampal input arising in the entorhinal cortex), inward hippocampal deformations found in clinical populations have been previously taken as probable consequence of disease-mediated reductions in nerve fibers in Alzheimer's disease (Li et al., 2007) and schizophrenia (Mamah et al., 2012) which disrupt cognitive function. Hippocampal deterioration is present in nonpathological aging, making it reasonable to apply these inferences about hippocampal deformations and basic neurobiology to the current findings relating to inward deformations. However, this would lead us to infer that outward deformations may reflect resilience, whereas we found outward deformations to be associated with poorer processing speed at bilateral subiculum. One speculative interpretation may be that this reflects a relative preservation of areas that exert inhibitory signaling in processing speed-related functions (e.g., Lipski and Grace, 2013), though direct data linking hippocampal morphology and neurobiology should be a priority for future research.

Despite the fact that childhood intelligence did not predict hippocampal shape deformations in older age, the nominal uncorrected associations between these deformations and age 11 IQ were observed in the same regions that were also associated (non-significantly) with fluid intelligence in older age. A smaller study (n = 137) on individuals from 18 to 86 years of age also showed similar result (Voineskos et al., 2015) but measured subfield volumes rather than morphology. Another study of similar sample size (n = 110), evaluated the correlation between educational attainment in youth, and hippocampal shape deformations reported significant associations in the same locations as our study (Tang et al., 2016). This may indicate that there is an inner tendency of certain hippocampal regions to be deformed inward or outward with respect to a medial shape depending on people's intelligence and independent of age, although a direct association does not seem to exist.

The association between spatial memory ability and the hippocampus receives broad support from previous studies (Colom et al., 2013, van Petten, 2004). In particular, spatial memory has previously been related to the volume of the right hippocampal tail (Chen et al., 2010). However, it is important to observe that the Wechsler Spatial Span task administered here does not provide an index of pure allocentric spatial ability, which is well studied with respect to hippocampal functioning (Ekstrom et al., 2014). Rather spatial span is a complex task that may employ multiple or different frames of reference (e.g., Bernardis and Shallice, 2011), and the results here should be interpreted in that context. The finding that measures of short term, working memory, and verbal memory was not associated with hippocampal shape after correction for multiple comparisons may be considered unsurprising. However, prior work indicates that the hippocampus may not be relevant for some processes such as memory binding (Baddeley et al., 2010) nor for verbal processing (Colom et al., 2013), though there is functional and volumetric evidence for the involvement of the hippocampus in immediate and delayed verbal memory (de Chastelaine et al., 2016). It should be noted that across all memory tests (and also within the general memory score), that there were associations in consistent directions with the subiculum and in clusters at the head and tail of the CA1 region. However, these associations did not survive correction for multiple comparisons, and although FDR is considered a relatively liberal correction approach, it should be noted that it cannot account for the spatial relatedness of clustered peaks, which are relatively uncommon. Moreover, the inability to reliably detect effects of hippocampal shape on some memory tests could also be due to the relative good health of the study participants; this precludes a clear generalization of our findings to other populations, such as those with clinical neurodegenerative or neuropsychiatric conditions.

One question in a cohort of this age is to what extent the results reflect normal age-related variations in hippocampal shape as opposed to reflecting a proportion of subjects who may be in the earliest stages of dementia or other age-related neurodegeneration. The exclusion criteria utilized (MMSE score of less than 24 or existing diagnosis of dementia) may not capture participants either in the early or presymptomatic stages of disease. Despite the unavailability of biomarkers of Alzheimer's pathology (e.g., amyloid PET or CSF markers) in this cohort, the current literature suggests 20% or more asymptomatic individuals in this age group may have evidence of Alzheimer's pathology (Sperling et al., 2011). Studies on hippocampal morphology in Alzheimer's disease patients (Kim et al., 2015, Perrotin et al., 2015, Qiu et al., 2009, Sarazin et al., 2010, Shen et al., 2012) and individuals with MCI (Costafreda et al., 2011, Lim et al., 2012)—albeit using different shape modeling methods—show associations between different cognitive tests and hippocampal shape deformations in the same locations and directions (i.e., inward/outward a “median or mean” shape) as our study. Though our data had no extreme outliers, it is currently impossible to ascertain the number (likely a small minority) of presymptomatic individuals in the current cohort, and the degree to which any presymptoms have exerted leverage on our results. Such information will only be possible with continued follow-up and future data linkage with national health records. We therefore caution that our findings apply generally to currently non-demented, community-dwelling older adults, rather than exclusively to nonpathological aging.

This study has other limitations. First, we did not include measures of other brain regions. Thus, it is possible that hippocampal shape and processing speed, for example, are both related to other brain measures such as white matter microstructure, frontal lobe regions, or general brain atrophy, but that processing speed is not directly constrained by hippocampal shape per se. Future studies could focus on longitudinal data which examines change-change correlations in light of other brain MRI indices. Also, cross-sectional studies could examine hippocampal morphology in relation to other brain regions' morphology and/or microstructure to inform of possible associations and/or patterns on different populations. It should also be noted that the effect sizes for associations between morphology and cognitive abilities were generally modest. Although we were well-powered to detect these effects, it is possible that such effects may not be reliably detected in less well-powered settings. However, our findings in this healthy, self-selecting cohort are likely to be underestimates of population-level effect sizes, and we also note that morphological analysis estimates were of a greater magnitude than those for hippocampal volume. Furthermore, though shape analysis is a powerful tool to investigate small changes in the outer surface of the hippocampus and its subregions, inferences on inner hippocampal subfields such as the dentate gyrus cannot be made. Finally, further information on the relative contributions of hippocampal morphology and volume to cognitive abilities would benefit from direct comparisons with subfield volumes. However, as outlined in the introduction, their accurate delineation requires greater resolution and a higher field strength than is available here, and there remains no consensus on a single segmentation protocol (Adler et al., 2014, Shing et al., 2011).

Among the study's strengths is the narrow age range (which largely rules out the confounding effect of chronological age), and control for other important confounds such as vascular risk, and the large sample size. The hippocampal masks on which the morphological analysis was based were each visually inspected and manually edited to ensure high quality. The hippocampal modeling method employed here was validated specifically on older individuals who were experiencing nonpathological aging, MCI, and AD patients (Kim et al., 2015). We also used a cohort-specific template to minimize the potential for registration errors and ensured the hippocampal shape modeling could accurately reproduce the shape details and correct for the rough boundaries of the binary masks. This enabled us to demonstrate a complex pattern of hippocampal deformations across a wide range of well-characterized cognitive abilities in older age.

To the best of our knowledge, this is the first study on a large older and cognitively normal population exploring the associations between hippocampal morphology and cognitive functions. Nevertheless, the deformation patterns found are similar to those presented by other studies that explored hippocampal morphology in cognitively different groups of individuals with ages ranging from middle to late adulthood (Perrotin et al., 2015, Qiu et al., 2009, Voineskos et al., 2015). Asymmetry in the patterns obtained for left and right hippocampi was also a corroborative result. This asymmetry has been previously reported not only for the hippocampus but also for the whole temporal region in MCI and AD patients (Moretti et al., 2012). Overall, this study indicates that a consistent pattern of both inward and outward hippocampal deformations in certain regions is associated with specific cognitive functions in older age and suggests that complex shape-based hippocampal analyses may provide valuable information beyond gross volumetry.

## Disclosure statement

The authors have no conflicts of interest to disclose.

## Figures and Tables

**Table 1:** Sample characteristics (n = 654)

Age	Mean (SD) y	72.50 (0.71)
Left hippocampal volume	Mean (SD) mm^3^	3333.80 (456.66)
Right hippocampal volume	Mean (SD) mm^3^	3095.29 (462.13)
Total brain volume	Mean (SD) mm^3^	991,524.60 (89,528.66)
Logical memory total score (I + II)	Mean (SD)	74.90 (17.86)
Verbal paired associates total score (I + II)	Mean (SD)	27.37 (9.61)
Spatial span total	Mean (SD)	14.79 (2.71)
Letter-number sequencing	Mean (SD)	11.03 (3.01)
Digit span backwards	Mean (SD)	7.90 (2.31)
g	Mean (SD)	0.05 (0.98)
g-speed	Mean (SD)	0.03 (0.98)
g-memory	Mean (SD)	0.03 (1.02)
Age 11 IQ	Mean (SD)	101.05 (13.88)
Body mass index	Mean (SD) (kg/m^2^)	27.89 (4.38)
Diastolic blood pressure	Mean (SD) (mm Hg)	79.82 (9.44)
Systolic blood pressure	Mean (SD) (mm Hg)	146.81 (18.18)
IFFC-HbA_1c_	Mean (SD) (mmol/mol)	39.08 (7.84)
History of hypertension	n (%)	322 (49.24)
History of diabetes	n (%)	69 (10.55)
History of hypercholesterolemia	n (%)	275 (42.05)
History of cardiovascular disease	n (%)	179 (27.37)
Previous stroke (history or imaging)	n (%)	117 (17.89)
Previous smokers	n (%)	295 (45.11)
Current smokers	n (%)	51 (7.80)
