# Supplementary material for: Hippocampal morphology and cognitive functions in community-dwelling older people: the Lothian Birth Cohort 1936
Source: Neurobiol Aging. 2017 Apr;52:1–11. doi: 10.1016/j.neurobiolaging.2016.12.012 (PMC5364373; doi:10.1016/j.neurobiolaging.2016.12.012)
Supplement: Supplementary Tables 1–3 and Supplementary Figs. 1 and 2 [file mmc1.doc]

**Supplementary Material**

**Table S1.** Cognitive tests used to derive indices of the cognitive domains of g, g-speed and g-memory using principal component analysis (PCA).

| Domain | Tests | Loadings | % Variance |
| --- | --- | --- | --- |
| *g* | Symbol Search^a^, Digit Symbol^a^, Matrix Reasoning^a^, Letter-Number Sequencing^a^, Digit Backwards^b^, Block Design^a^. | >0.419 | 51.19 |
| g-speed | Symbol Search^a^, Digit Symbol^a^, Choice Reaction Time^c^, Simple Reaction Time^c^, Inspection Time^c^. | >.407 | 52.51 |
| g-memory | Logical Memory^b^, Verbal Paired Associates^b^, Spatial Span^b^, Digit Backwards^b^, Letter Number Sequencing^a^. | >.488 | 39.21 |

*Note.* ^a^ WAIS III^UK^, ^b^ WMS III^UK^, ^c^ see Deary *et al.* (2007) for further details. % Variance denotes the proportion of variance explained by the first unrotated component of a PCA comprising the tests indicated.

**Table S2.** Correlations between hippocampal volumes (before and after adjusting for brain size), cognitive and health variables.

|  | Raw hippocampal volumes | | | Hippocampal volumes adjusted for brain size | | |
| --- | --- | --- | --- | --- | --- | --- |
|  | RHV (mm^3^) | LHV (mm^3^) | THV (mm^3^) | % RHV in BTV | % LHV in BTV | % THV in BTV |
| Spatial | 0.107^b^ | 0.132^c^ | 0.125^b^ | -0.046 | -0.009 | -0.029 |
| Logical Mem | 0.060 | 0.086^a^ | 0.077 | 0.029 | 0.063 | 0.050 |
| VPA | 0.012 | 0.059 | 0.046 | 0.022 | 0.069 | 0.049 |
| Digit Bckwds | 0.082^a^ | 0.099^a^ | 0.077 | 0.044 | 0.072 | 0.063 |
| LN Seq | 0.081^a^ | 0.092^a^ | 0.085^a^ | 0.022 | 0.045 | 0.036 |
| *g* | 0.125^b^ | 0.106^b^ | 0.111^b^ | -0.014 | -0.016 | -0.016 |
| Speed | 0.086 | 0.064 | 0.075 | -0.046 | -0.052 | -0.053 |
| Memory | 0.076 | 0.119^b^ | 0.101^a^ | 0.018 | 0.071 | 0.049 |
| Age 11 IQ | 0.035 | 0.051 | 0.038 | 0.032 | 0.004 | 0.019 |
| BMI | 0.078^a^ | 0.065 | 0.063 | 0.121^b^ | 0.101^a^ | 0.119^b^ |
| Diastolic BP | 0.059 | 0.072 | 0.046 | 0.033 | 0.046 | 0.043 |
| Systolic BP | 0.049 | 0.023 | 0.029 | 0.051 | 0.021 | 0.038 |
| HbA1c | 0.024 | 0.001 | 0.016 | 0.090^a^ | 0.057 | 0.078 |

*Note:* Pearson’s *r* reported. ^a^ *p*<0.05, ^b^ *p*<0.01, ^c^ *p*<0.001. Logical Mem = Total of Logical Memory I and II, VPA = Verbal Paired Associates total of I and II, BMI = Body Mass Index, BP = Blood Pressure, RHV = Right Hippocampal Volume, LHV = Left Hippocampal Volume, BTV = Total Brain Tissue Volume

**Table S3.** Linear regressions between hippocampal volume and cognitive tests and domains, correcting for age at scan, gender and health factors.

|  | Raw hippocampal volumes | | | Hippocampal volumes adjusted for brain size | | |
| --- | --- | --- | --- | --- | --- | --- |
|  | RHV | LHV | THV | % RHV in BTV | % LHV in BTV | % THV in BTV |
| ***Memory Subtests*** | |  |  |  |  |  |
| Spatial | 0.067 (0.110) | 0.098 (0.018) | 0.089 (0.034) | -0.039 (0.326) | -0.002 (0.956) | -0.022 (0.580) |
| Verbal | 0.079 (0.063) | **0.115 (0.006)** | 0.105 (0.014) | 0.009 (0.814) | 0.054 (0.190) | 0.034 (0.399) |
| Digit Bckwds | **0.111 (0.009)** | **0.120 (0.004)** | **0.125 (0.004)** | 0.036 (0.369) | 0.061 (0.140) | 0.053 (0.199) |
| LN Seq | 0.102 (0.016) | **0.108 (0.010)** | 0.113 (0.008) | 0.020 (0.619) | 0.041 (0.321) | 0.033 (0.419) |
|  |  |  |  |  |  |  |
| ***Domains*** |  |  |  |  |  |  |
| *g* | **0.133 (0.001)** | 0.101 (0.014) | **0.126 (0.002)** | -0.015 (0.694) | -0.027 (0.496) | -0.023 (0.561) |
| g-speed | 0.088 (0.031) | 0.052 (0.201) | 0.075 (0.067) | -0.056 (0.153) | -0.074 (0.063) | -0.070 (0.076) |
| g-memory | 0.108 (0.013) | **0.145 (0.001)** | **0.137 (0.002)** | 0.007 (0.863) | 0.056 (0.182) | 0.035 (0.410) |
| Age 11 IQ | 0.077 (0.071) | 0.082 (0.054) | 0.086 (0.046) | -0.039 (0.334) | -0.021 (0.618) | -0.033 (0.431) |

*Note.* Standardised β *(p* values*)* reported. RHV = Right hippocampal volume (mm^3^), LHV = Left hippocampal volume (mm^3^), THV = Total hippocampal volume (mm^3^), BTV = Total brain tissue volume, LN Seq = Letter Number Sequencing, g = general cognitive function (i.e. fluid intelligence). Bold typeface indicates significant following FDR correction.


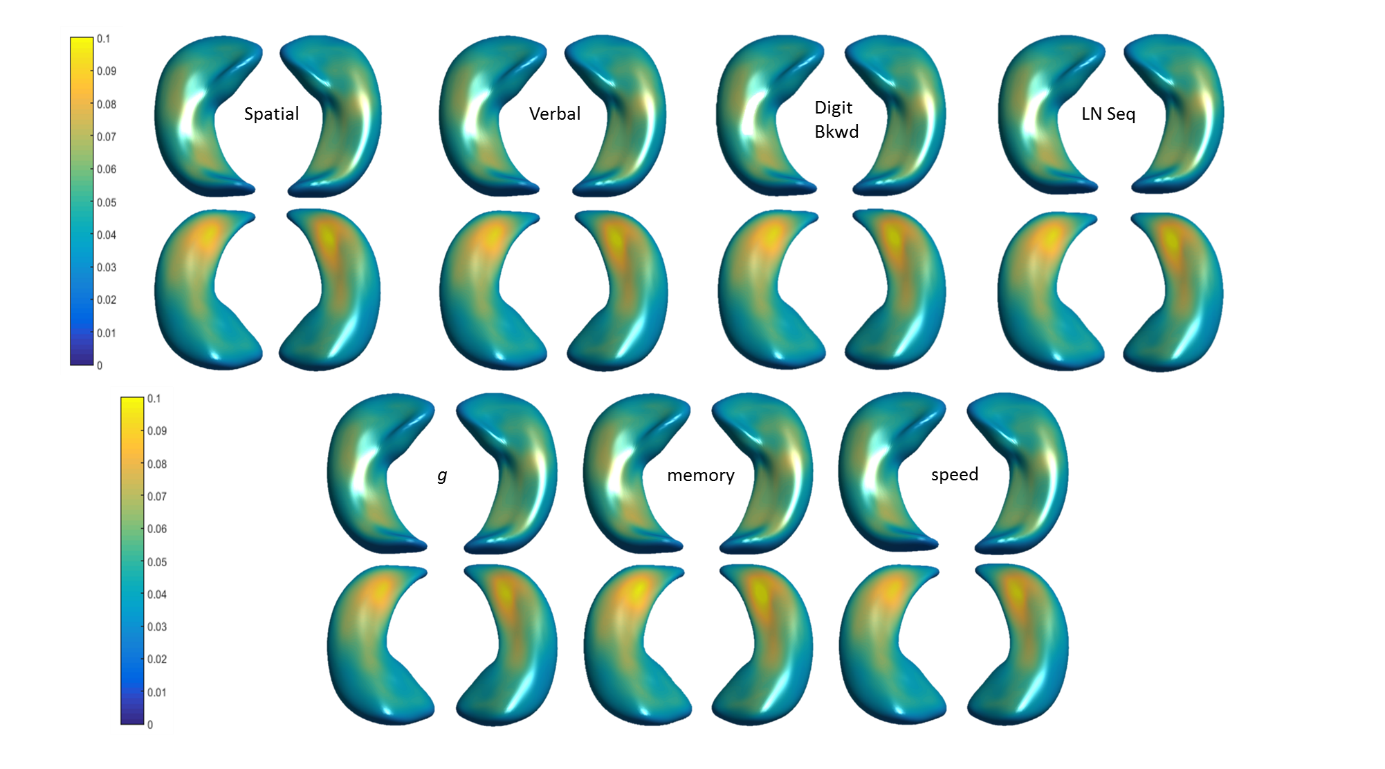


*Supplementary Figure 1.* Standard errors (<0.10) are displayed across the mesh surface for each regression model at each vertex between cognitive ability and hippocampal shape deformation, corrected for age, sex and vascular risk (corresponding to Figures 2 and 4).

*
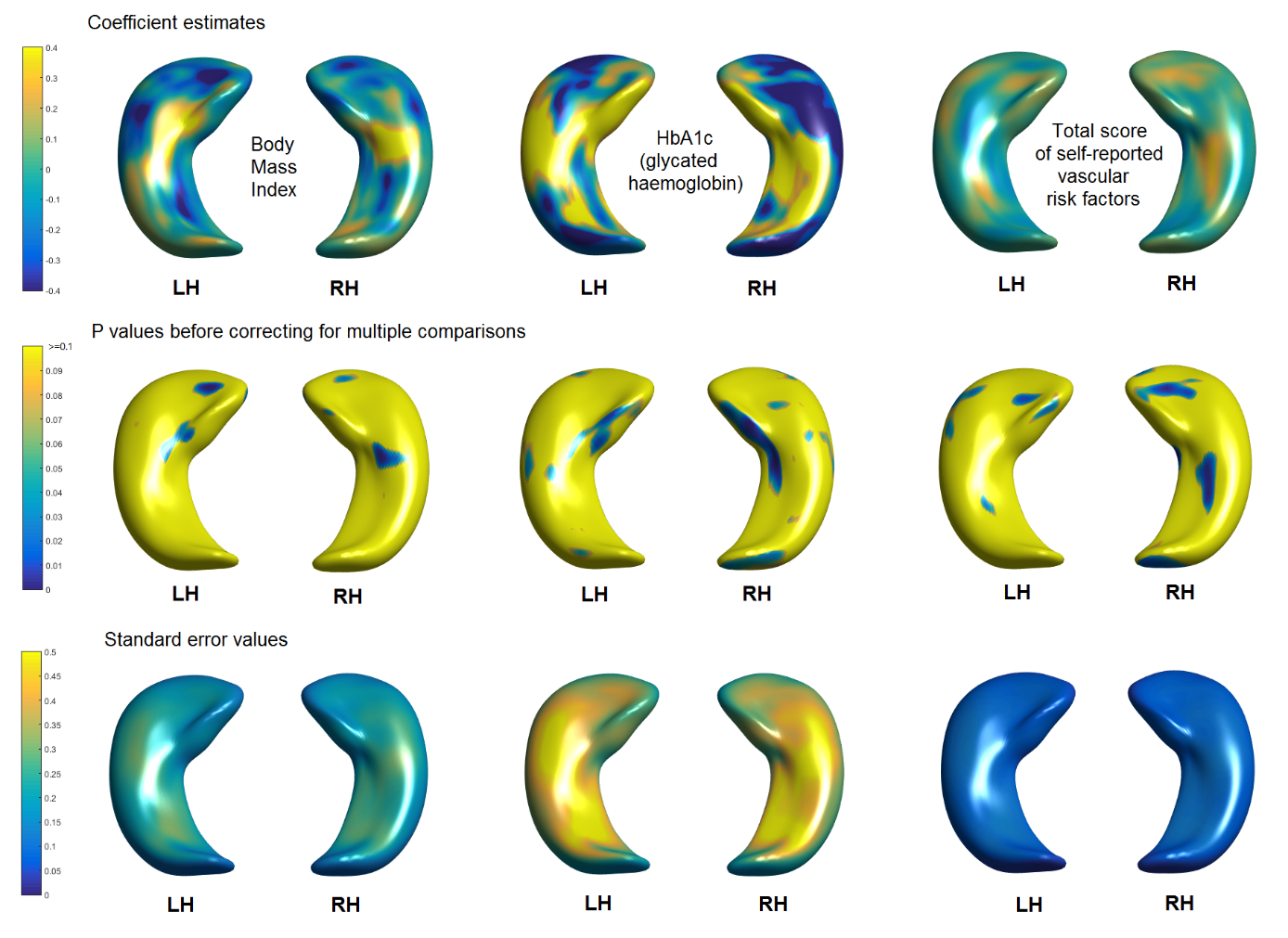
Supplementary Figure 2.* Unstandardised estimates of associations between hippocampal shape deformations and vascular risk factors (upper row), *p*-values prior to FDR correction (middle row) and standard errors (bottom row). The model that explores associations between hippocampal deformations and glycated haemoglobin only fit in regions at the hippocampal head and extreme of the tail (see standard errors). LH: left hippocampi, RH: right hippocampi.
